# Supplementary material for: The effect of early pregnancy ALT elevation on neonatal birth weight: The mediating role of gestational diabetes mellitus
Source: PLoS One. 2025 May 14;20(5):e0322581. doi: 10.1371/journal.pone.0322581 (PMC12077732; doi:10.1371/journal.pone.0322581)
Supplement: S1 Tables — (DOCX) [file pone.0322581.s001.docx]

**supporting information**

**S1 Tables. Shapiro-Wilk normality test for continuous variables grouped according to LGA**

|  | Statistic | Degrees of Freedom (df) | P |
| --- | --- | --- | --- |
| age, year | 0.966 | 1274 | ＜0.001 |
| pregnancy weight gain, kg | 0.993 | 1274 | ＜0.001 |
| delivery gestational week, week | 0.792 | 1274 | ＜0.001 |
