# Supplementary material for: The effect of early pregnancy ALT elevation on neonatal birth weight: The mediating role of gestational diabetes mellitus
Source: PLoS One. 2025 May 14;20(5):e0322581. doi: 10.1371/journal.pone.0322581 (PMC12077732; doi:10.1371/journal.pone.0322581)
Supplement: Attachment — Submitted filename: renamed_c4d20.docx [file pone.0322581.s002.docx]

退稿

Dear Editors：

Thank you very much for your invitation! The following are the comments regarding the manuscript PONE-D-24-42399 ‘The effect of elevated ALT in early pregnancy on neonatal birth weight: the mediating role of gestational diabetes mellitus’.

Title and abstract

1. In your opinion, how does the title and abstract effectively capture the content and focus of the manuscript? Please suggest any improvement if needed.

Title: Write properly

Abstract: Increase the contents of research significance of this study

In conclusion part of the abstract, please not only repeated the results, but should focus on and advise medical providers to increase their policy measures on the research results.

2. Did you find that the background and information provided was sufficient to understand the research? Would you have any other suggestions?

I would rather like to see some background statistics on the prevalence of ALT in China or the prevalence of the study, to give the reader some background information about the research background. I think this introduction requires more review of the literature.

Materials and Methods

3. Based on your expertise, how will you assess the clarity of the methods used in your study?

What are the tests for exposure variables?

Why did this study take the diagnosis of blood glucose at 13 weeks rather than using the criteria for gestational diabetes, It suggested to add literature here, how do you control all the confounding factors in the study? Each variable will need to be supported by the literature. Should the covariates also include pregnant women with dyslipidemia?

How is the sample size calculated?

When was your informed consent form signed? How to sign it?

Statistical analysis of the mediation effect model needs more literature to support.

4. If applicable, please share your views on the novelty of the study findings.

The results are very well presented

discuss

5. Are the findings described by the authors relevant to the results? From your perspective, are the results related to the objectives of the study and the broader research context?

The results are well discussed and suggest satisfactory supportive reasoning, similar studies are supported and the authors try to find supportive studies in each significant variable, but I still needed that the introduction should support all the issues discussed.

6. In your opinion, how do these conclusions fit with the research results?

This manuscript is written clearly and all limitations are highlighted, but in this study it is a single-center, retrospective design that may introduce bias. Insufficient control of confounding factors to fully control the confounding factors affecting pregnancy-related ALT levels and GDM, multicenter studies can increase the sample diversity and reduce the bias caused by factors such as geography, making the results more universal.

Charts and tables

7. If the author provides the charts, are they clear and clearly visible? Are these numbers unnecessarily modified?

Column a diagram of the study steps and the intervention for each step so that the reader understands the entire study process.

8. Is this statistical analysis suitable for this study?

Yes

9. Are these references relevant to this study?

Yes

10. Is the referenced style correct?

Yes

11. Are you concerned about the similarity with other articles published by the same author or any other questions?

no-one

Competitive interests

12. Did any authors competing interests cause concern about the validity of the study, namely, did the authors competing interests generate competing interests in the reporting of the results and conclusions?

not have

Best regards,

Yours sincerely

Dr Zhai

Tuesday, December 31,2024
