# Supplementary material for: The effect of early pregnancy ALT elevation on neonatal birth weight: The mediating role of gestational diabetes mellitus
Source: PLoS One. 2025 May 14;20(5):e0322581. doi: 10.1371/journal.pone.0322581 (PMC12077732; doi:10.1371/journal.pone.0322581)
Supplement: Attachment — Submitted filename: Response to Reviewers.docx [file pone.0322581.s004.docx]

Dear Editor and Reviewers,

Thank you for the opportunity to revise our manuscript titled "The Effect of Early Pregnancy ALT Elevation on Neonatal Birth Weight: The Mediating Role of Gestational Diabetes Mellitus" (manuscript number: PONE-D-24-42399). We are truly grateful for the insightful and constructive feedback provided by the reviewers, which has significantly contributed to enhancing the quality and clarity of our work.

We would also like to make a humble request regarding authorship. Dr. Zhou Xu, who was the data owner at Sichuan Jinxin Xinan Women and Children’s Hospital and made substantial contributions to manuscript design, drafting, and revision, was unintentionally omitted as a co-first author. We deeply regret this oversight and would like to amend the manuscript to reflect his rightful contribution. Similarly, Prof. Yu-Bin Ding should be designated as a co-corresponding author, as his contributions are integral to the study. After reassessing the contributions, we concluded that both Dr. Zhou Xu and Prof. Yu-Bin Ding's roles in the research are of equal significance. All authors have been consulted, and we have unanimously agreed to make this adjustment.

Attached, please find our point-by-point responses to each reviewer comment. We have carefully addressed all suggestions and revisions, and we hope that the revised manuscript meets the journal’s standards for publication.

We welcome any further comments and sincerely appreciate the time and effort the reviewers have dedicated to evaluating our work. If additional documentation or information is required for the authorship change, please do not hesitate to let us know.

Thank you again for your consideration and support.

Sincerely,

Zhao-Hui Zhong, Prof.

Department of Obstetrics and Gynecology, Women and Children’s Hospital of Chongqing Medical University

Joint International Research Laboratory of Reproduction and Development of the Ministry of Education of China

School of Public Health, Chongqing Medical University, Chongqing 400016, China

**Point-to-point response to reviewers and editors**

**Editors #**

1.Please ensure that your manuscript meets PLOS ONE's style requirements, including those for file naming.

**Response:**

Thank you for your feedback and guidance. We have ensured that the manuscript adheres to PLOS ONE’s formatting requirements, using the provided templates and adjusting the title, author information, and file naming accordingly.

2.Thank you for stating the following financial disclosure:

“This work was supported by the National Key Research and Development Program of China (no: 2023YFC2705900) and the Open Fund of Chongqing Maternal and Child Disease Control and Public Health Research Center (no: CQFYSJ01001).”

Please state what role the funders took in the study. If the funders had no role, please state: "The funders had no role in study design, data collection and analysis, decision to publish, or preparation of the manuscript."

If this statement is not correct you must amend it as needed.

Please include this amended Role of Funder statement in your cover letter; we will change the online submission form on your behalf.

**Response:**

Thank you for your feedback regarding the financial disclosure statement. We have revised the statement as follows:

“This work was supported by the Open Fund of Chongqing Maternal and Child Disease Control and Public Health Research Center (no: CQFYSJ01001). The funders had no role in study design, data collection and analysis, decision to publish, or preparation of the manuscript.”

Please update the online submission form accordingly.

3.In this instance it seems there may be acceptable restrictions in place that prevent the public sharing of your minimal data. However, in line with our goal of ensuring long-term data availability to all interested researchers, PLOS’ Data Policy states that authors cannot be the sole named individuals responsible for ensuring data access

(http://journals.plos.org/plosone/s/data-availability#loc-acceptable-data-sharing-methods).

Data requests to a non-author institutional point of contact, such as a data access or ethics committee, helps guarantee long term stability and availability of data. Providing interested researchers with a durable point of contact ensures data will be accessible even if an author changes email addresses, institutions, or becomes unavailable to answer requests.

Before we proceed with your manuscript, please also provide non-author contact information (phone/email/hyperlink) for a data access committee, ethics committee, or other institutional body to which data requests may be sent. If no institutional body is available to respond to requests for your minimal data, please consider if there any institutional representatives who did not collaborate in the study, and are not listed as authors on the manuscript, who would be able to hold the data and respond to external requests for data access? If so, please provide their contact information (i.e., email address). Please also provide details on how you will ensure persistent or long-term data storage and availability.

**Response:**

Thank you for your valuable feedback and guidance. We fully understand the importance of ensuring long-term data availability in compliance with the journal's data policy. Unfortunately, we do not have non-author contact information (phone/email/hyperlink) available for direct data access requests. To address this, we have identified institutional representatives who were not involved in the study and are not listed as authors on the manuscript, but who can hold the data and respond to external data access requests. The contact information is as follows: email: pinyi.chen@cqmu.edu.cn; Phone: +86 23 68485111; Fax: +86 23 63846904].

4.Please amend either the title on the online submission form (via Edit Submission) or the title in the manuscript so that they are identical.

**Response:**

Thank you for your comment. We have made the necessary amendment to ensure that the title in the manuscript exactly matches the one on the online submission form, as requested.

5. Your ethics statement should only appear in the Methods section of your manuscript. If your ethics statement is written in any section besides the Methods, please delete it from any other section.

**Response:**

Thank you for your feedback and guidance. We have moved the ethics statement to the methods section and removed the ethics statements in other sections.

6. Please include a separate caption for each figure in your manuscript.

**Response:**

Thank you for your feedback and guidance. Each figure now has a separate, clearly written caption.

7.Please include your tables as part of your main manuscript and remove the individual files. Please note that supplementary tables (should remain/ be uploaded) as separate "supporting information" files.

**Response:**

Thank you for your feedback and guidance. All tables have been incorporated into the main manuscript, and supplementary tables have been uploaded as separate supporting information files.

**Reviewer 1#**

**Comment 1:**

The results section would benefit from clearer organization of the mediation analysis findings.

**Response:**

Thank you for your valuable suggestion. In response, we have reorganized the mediation analysis findings in the results section to improve clarity. Specifically, we have added subheadings to differentiate between "Direct Effects of ALT," "Indirect Effects via GDM," and "Proportion of Mediation." Each section now presents the mediation effects in a consistent format, including β values, confidence intervals, and percentages of mediation for both the natural conception and assisted reproduction groups. This restructuring should provide a clearer understanding of the results. Please refer to the revised tables and the corresponding description of results.

**Comment 2:**

Methods section could benefit from more detail about the mediation analysis methodology.

**Response:**

We appreciate this feedback. To address this, we will: Provide additional details on the mediation analysis methodology, including the statistical model used (e.g., causal steps approach, bootstrapping, or structural equation modeling). Include a description of the software and packages used for mediation analysis.

Please refer to the revised

**Comment 3:**

The Methods section should clarify the power calculation or sample size determination.

**Response:**

Thank you for the valuable feedback from the reviewer. Regarding the calculation of sample size, we estimated it based on logistic regression analysis and mediation analysis, and ultimately determined that the minimum sample size required for this study is 412.In practice, we included 12,057 research subjects, which fully satisfies the sample size requirement. We have added a section on "Sample Size Calculation" in the Methods section.

**Comment 4:**

The methods section lacks information about the methods utilized to evaluate ALT and glucose levels; the cut-off values and their coefficient of variability need to be specified in detail for reproducibility.

**Response:**

Thank you for your helpful comment. In response, we have now included detailed information on the laboratory methods used for measuring ALT and glucose levels in the Methods section. Specifically, we have provided the cut-off values and the coefficient of variability for these assays to ensure reproducibility of our findings. This additional information should help clarify the methodology used for these measurements.

**Comment 5:**

Mann-Whitney U test is used for non-parametric variables and should be given by median and IQR or min-max. If the values are given by mean and SD, the variables must be normally distributed, and differences for the means are calculated by Student's t-test. Please give information about the data distribution.

**Response:**

Thank you for the valuable feedback. We have performed normality tests (Shapiro-Wilk) for all continuous variables. For non-normally distributed variables, we reported medians and IQRs and used the Mann-Whitney U test. For normally distributed variables, we used means ± SD and applied t-tests.

These changes are now reflected in the Statistical Analysis section and Table 1. A supplementary table has been added to the results section, displaying the Shapiro Wilk normality test for each variable.

**Reviewer 2#**

**Comment 1:**

In conclusion part of the abstract, please not only repeated the results, but should focus on and advise medical providers to increase their policy measures on the research results.

**Response:**

Thank you for the constructive comments from the reviewer. We have added policy recommendations for healthcare providers in the conclusion section of the abstract, which not only emphasizes the value of the research but also promotes its practical application.

**Comment 2:**

I would rather like to see some background statistics on the prevalence of ALT in China or the prevalence of the study, to give the reader some background information about the research background. I think this introduction requires more review of the literature.

**Response:**

Thank you for your valuable suggestion. We have now incorporated relevant data on the prevalence of ALT abnormalities in China and referenced studies that highlight the significance of ALT levels in pregnancy.

**Comment 3:**

What are the tests for exposure variables?

**Response:**

Thank you for your question. The exposure variable, elevated ALT levels, was assessed using enzymatic methods, following established clinical laboratory protocols. We have now clarified this in the Methods section of the manuscript to provide a more detailed explanation of the testing procedure.

**Comment 4:**

Why did this study take the diagnosis of blood glucose at 13 weeks rather than using the criteria for gestational diabetes, It suggested to add literature here.

**Response:**

Thank you for your valuable feedback. In this study, all patients were screened for GDM in strict accordance with the Diagnosis and Treatment Guidelines for Gestational Hyperglycemia (2022). At 24-28 weeks of pregnancy, a 75 g oral glucose tolerance test (OGTT) was performed: the blood glucose thresholds on an empty stomach and 1 hour and 2 hours after oral glucose were 5.1, 10.0, and 8.5 mmol/L, respectively. If the blood glucose value reached or exceeded the specified criteria at any time point, it was diagnosed as GDM.

The aim of this study is to explore whether gestational diabetes mellitus (GDM) acts as a mediator in the relationship between early pregnancy ALT levels and neonatal birth weight. Since ALT levels were measured at 11-13 weeks of pregnancy, we wanted to emphasize that the GDM screening was conducted after the ALT measurement. This is why we stated that blood glucose was tested after 13 weeks. We apologize for any confusion caused by the lack of clarity in our original expression and have made the necessary revisions in the manuscript.

**Comment 5:**

How do you control all the confounding factors in the study? Each variable will need to be supported by the literature. Should the covariates also include pregnant women with dyslipidemia?

**Response:**

Thank you for your insightful comment regarding the control of confounding factors in the study. We recognize the importance of controlling for potential confounders to ensure the validity of our results.

In this study, the following methods were used to effectively control confounding factors and ensure the accuracy and reliability of the results:

1.Selection of Relevant Covariates: Based on the literature, multiple variables that could influence pregnancy outcomes (such as maternal age, pre-pregnancy BMI, gestational weight gain, gestational hypertension, thyroid disorders, etc.) were selected as covariates to control for potential confounding effects.

2.Multivariable Regression Analysis: A multivariable regression analysis was used to simultaneously account for multiple potential factors that could influence the outcome, effectively controlling for confounding factors and ensuring a more accurate assessment of the relationship between ALT, GDM, and LGA.

3.Mediation Analysis: Mediation analysis was conducted to explore whether GDM acts as a mediator in the relationship between elevated ALT and LGA, controlling for both direct and indirect effects, and revealing the complex causal relationships.

4.Stratified Analysis: Stratified Analysis can be used to further refine the results and ensure accurate estimation of whether GDM plays a mediating role in the relationship between ALT elevation and LGA in different subgroups.

We controlled for the following factors:

1.Maternal age:

Maternal age is a well-established factor influencing pregnancy outcomes. Studies have shown that advanced maternal age is associated with an increased risk of GDM and larger newborns, and it may also affect ALT levels.

Reference:

- Londero AP, Rossetti E, Pittini C, et al. Maternal age and the risk of adverse pregnancy outcomes: a retrospective cohort study. BMC PREGNANCY CHILDB. [Journal Article]. 2019 2019-7-23;19(1):261. doi: 10.1186/s12884-019-2400-x
- Kim EH, Lee J, Lee SA, et al. Impact of Maternal Age on Singleton Pregnancy Outcomes in Primiparous Women in South Korea. J CLIN MED. [Journal Article]. 2022 2022-2-12;11(4). doi: 10.3390/jcm11040969

2.Pre-pregnancy BMI:

Pre-pregnancy BMI has been consistently identified as a key factor influencing gestational weight gain, GDM development, and neonatal birth weight. Higher pre-pregnancy BMI is associated with abnormal liver function during pregnancy, as obesity may lead to liver fat deposition and metabolic abnormalities.

Reference:

- Liu P, Xu L, Wang Y, et al. Association between perinatal outcomes and maternal pre-pregnancy body mass index. OBES REV. [Journal Article; Meta-Analysis; Review]. 2016 2016-11-1;17(11):1091-102. doi: 10.1111/obr.12455
- Li M, Wang L, Du Z, et al. Joint effect of maternal pre-pregnancy body mass index and folic acid supplements on gestational diabetes mellitus risk: a prospective cohort study. BMC PREGNANCY CHILDB. [Journal Article]. 2023 2023-3-23;23(1):202. doi: 10.1186/s12884-023-05510-y

3.Gestational Weight Gain:

Excessive weight gain during pregnancy is associated with increased liver enzymes during pregnancy, diabetes in pregnancy and newborn birth weight.

Reference:

- Shekaili H, Hashmi IA, Omari OA. Gestational weight gain and its association with maternal and neonatal outcomes among pregnant women with normal pre-pregnancy body mass index from a Middle Eastern country. MIDWIFERY. [Journal Article]. 2024 2024-3-1;130:103929. doi: 10.1016/j.midw.2024.103929

4.whether it is the first pregnancy:

Parity can influence pregnancy outcomes, as primiparous women may face a higher risk of certain complications.

Reference:

- Dai J, Shi Y, Wu Y, et al. The interaction between age and parity on adverse pregnancy and neonatal outcomes. FRONT MED-LAUSANNE. [Journal Article]. 2023 2023-1-20;10:1056064. doi: 10.3389/fmed.2023.1056064

5.Assisted reproductive technology:

Due to the influence of hormones, ART surgery is related to gestational diabetes and newborn birth weight.

Reference:

- Bianchi C, Brocchi A, Baronti W, et al. Assisted reproductive technology, risk of gestational diabetes, and perinatal outcomes in singleton pregnancies. DIABETES-METAB RES. [Journal Article]. 2023 2023-7-1;39(5):e3625. doi: 10.1002/dmrr.3625

6.Delivery gestational week:

The gestational age during delivery can affect the birth weight of newborns. Premature and full-term delivery have different risks for birth weight, so it is necessary to control this variable.

7.Thyroid Disorders:

Thyroid diseases, such as hypothyroidism and hyperthyroidism, are known to impact pregnancy outcomes, including fetal growth.

Reference:

- Thyroid Disease in Pregnancy: ACOG Practice Bulletin, Number 223. OBSTET GYNECOL. [Journal Article; Practice Guideline]. 2020 2020-6-1;135(6):e261-74. doi: 10.1097/AOG.0000000000003893

8.Gestational Hypertension and Preeclampsia:

Both gestational hypertension and preeclampsia have been linked to an increased risk of adverse neonatal outcomes, including LGA and SGA. Pregnancy induced hypertension syndrome is one of the common causes of abnormal liver function during pregnancy.

Reference:

- Zhang B, Chen X, Yang C, et al. Effects of hypertensive disorders of pregnancy on the complications in very low birth weight neonates. HYPERTENS PREGNANCY. [Journal Article]. 2024 2024-12-1;43(1):2314576. doi: 10.1080/10641955.2024.2314576
- Bokslag A, van Weissenbruch M, Mol BW, et al. Preeclampsia; short and long-term consequences for mother and neonate. EARLY HUM DEV. [Journal Article; Review]. 2016 2016-11-1;102:47-50. doi: 10.1016/j.earlhumdev.2016.09.007

9.Pre-pregnancy diabetes:

Women with pre-pregnancy diabetes are at a higher risk of developing GDM and giving birth to LGA infants.

Reference:

- Ray JG, Vermeulen MJ, Shapiro JL, et al. Maternal and neonatal outcomes in pregestational and gestational diabetes mellitus, and the influence of maternal obesity and weight gain: the DEPOSIT study. Diabetes Endocrine Pregnancy Outcome Study in Toronto. QJM-INT J MED. [Comparative Study; Journal Article; Research Support, Non-U.S. Gov't]. 2001 2001-7-1;94(7):347-56. doi: 10.1093/qjmed/94.7.347

10.Intrahepatic Cholestasis of Pregnancy:

Pregnancy induced intrahepatic cholestasis is associated with an increased risk of adverse outcomes in newborns, and is one of the main causes of liver dysfunction during pregnancy.

Reference:

- Wikström SE, Marschall HU, Ludvigsson JF, et al. Intrahepatic cholestasis of pregnancy and associated adverse pregnancy and fetal outcomes: a 12-year population-based cohort study. BJOG-INT J OBSTET GY. [Journal Article; Research Support, Non-U.S. Gov't]. 2013 2013-5-1;120(6):717-23. doi: 10.1111/1471-0528.12174

Regarding your suggestion to include “dyslipidemia” as a covariate, thank you for your valuable suggestion. We considered the potential impact of dyslipidemia during the study design process, but current literature does not provide conclusive evidence that dyslipidemia directly affects ALT levels during pregnancy. Furthermore, in similar studies (such as those examining the relationship between ALT levels and neonatal birth weight), dyslipidemia has not been included as a covariate. Therefore, we did not include it as a confounding factor in our study. In addition, our preliminary analysis showed that dyslipidemia did not exhibit multicollinearity with other covariates, indicating that it would not cause significant bias in the results of our model. Thus, we believe that dyslipidemia does not constitute a confounding factor in this study.

We highly value your suggestion and are willing to further explore the potential impact of dyslipidemia in future research. If there is relevant evidence to support its importance as a confounding factor, we will also actively include supplementary analysis to verify the robustness of the research results.

- Yarrington CD, Cantonwine DE, Seely EW, et al. The association of early unexplained elevated alanine aminotransferase with large-for-gestational-age birthweight. AM J OBSTET GYNECOL. [Journal Article]. 2016 2016-10-1;215(4):471-4. doi: 10.1016/j.ajog.2016.04.051

**Comment 6:**

How is the sample size calculated?

**Response:**

Thank you for the valuable feedback from the reviewer. Regarding the calculation of sample size, we estimated it based on logistic regression analysis and mediation analysis, and ultimately determined that the minimum sample size required for this study is 412. In practice, we included 12,057 research subjects, which fully satisfies the sample size requirement.

We have added a section on "Sample Size Calculation" in the Methods section as well as the cited paper.

**Comment 7:**

When was your informed consent form signed? How to sign it?

**Response:**

Thank you for raising this important point. We apologize for the confusion in our previous description. To clarify, all patient data were anonymized to protect privacy and confidentiality. The ethics committees waived the requirement for written informed consent due to the minimal risk associated with using de-identified data. We have updated the manuscript to more clearly explain this process, and we have made corresponding modifications in the methods section to ensure clarity.

**Comment 8:**

Statistical analysis of the mediation effect model needs more literature to support.

**Response:**

Thank you for your constructive feedback. To address this issue, we have expanded the statistical analysis section by adding references to strengthen the theoretical foundation of our mediation analysis. These references provide detailed guidance for the causal step method and non parametric bootstrap method used in our research.

**Comment 9:**

The results are well discussed and suggest satisfactory supportive reasoning, similar studies are supported and the authors try to find supportive studies in each significant variable, but I still needed that the introduction should support all the issues discussed.

**Response:**

We appreciate your constructive feedback. In response to your comment, we have revised the introduction to better contextualize the key issues addressed in the study. Specifically, we have added more references and expanded on the background related to the association between ALT levels, gestational diabetes mellitus (GDM), and their impact on neonatal outcomes, including large for gestational age (LGA) infants. This revision ensures that the introduction adequately supports the variables discussed in the subsequent sections of the manuscript.

**Comment 10:**

This manuscript is written clearly and all limitations are highlighted, but in this study it is a single-center, retrospective design that may introduce bias. Insufficient control of confounding factors to fully control the confounding factors affecting pregnancy-related ALT levels and GDM, multicenter studies can increase the sample diversity and reduce the bias caused by factors such as geography, making the results more universal.

**Response:**

Thank you for your insightful comment. We appreciate your recognition of the clarity of the manuscript and the identification of limitations. We fully acknowledge that the study's single-center, retrospective design may introduce biases, and that the control of confounding factors could be more robust. As you rightly pointed out, the limited control of confounders may affect the generalizability of our findings. However, given the current constraints, conducting a multicenter study was not feasible. To address this limitation, we have suggested in the manuscript that future studies could benefit from a "multicenter, prospective design" to enhance the generalizability and reduce potential biases.

**Comment 11:**

Column a diagram of the study steps and the intervention for each step so that the reader understands the entire study process.

**Response:**

Thank you for the reviewer's suggestions. We have input a flowchart to better illustrate the research process and key steps.
