# Supplementary material for: The effect of early pregnancy ALT elevation on neonatal birth weight: The mediating role of gestational diabetes mellitus
Source: PLoS One. 2025 May 14;20(5):e0322581. doi: 10.1371/journal.pone.0322581 (PMC12077732; doi:10.1371/journal.pone.0322581)
Supplement: Attachment — Submitted filename: Response_to_Reviewers_auresp_2.docx [file pone.0322581.s005.docx]

Dear Editor and Reviewers,

Thank you for the opportunity to revise our manuscript titled "The Effect of Early Pregnancy ALT Elevation on Neonatal Birth Weight: The Mediating Role of Gestational Diabetes Mellitus" (manuscript number: PONE-D-24-42399R1). We are truly grateful for the insightful and constructive feedback provided by the reviewers, which has significantly contributed to enhancing the quality and clarity of our work.

Attached, please find our point-by-point responses to each reviewer comment. We have carefully addressed all suggestions and revisions, and we hope that the revised manuscript meets the journal’s standards for publication.

We welcome any further comments and sincerely appreciate the time and effort the reviewers have dedicated to evaluating our work.

Thank you again for your consideration and support.

Sincerely,

Zhao-Hui Zhong, Prof.

Department of Obstetrics and Gynecology, Women and Children’s Hospital of Chongqing Medical University

Joint International Research Laboratory of Reproduction and Development of the Ministry of Education of China

School of Public Health, Chongqing Medical University, Chongqing 400016, China

**Point-to-point response to reviewers and editors**

**Editors #**

1.Please review your reference list to ensure that it is complete and correct. If you have cited papers that have been retracted, please include the rationale for doing so in the manuscript text, or remove these references and replace them with relevant current references. Any changes to the reference list should be mentioned in the rebuttal letter that accompanies your revised manuscript. If you need to cite a retracted article, indicate the article’s retracted status in the References list and also include a citation and full reference for the retraction notice.

**Response:**

Thank you for your feedback. We have carefully reviewed all references cited in our manuscript and verified their status using PubMed and other relevant databases. We confirm that none of the references have been retracted. No changes were required in the reference list, but we appreciate the opportunity to ensure its accuracy. In response to the reviewer's comments, we have added an additional reference (Reference 31), which has also not been retracted, to further support our findings.

**Reviewer 1#**

**Response:**

We appreciate that Reviewer #1 considers all previous comments to have been adequately addressed. Thank you for your time and constructive feedback.

**Reviewer 3#**

**Comment 1:**

I check the manuscript and found that near to all parts of discussion, abstracts and other parts of the manuscript is written by AI. Is it in line with the journal policies?

**Response:**

Thank you for your attention to the language quality of our manuscript and for your important reminder regarding the use of AI tools. We fully understand the academic community's cautious attitude toward AI-generated content and would like to make a formal clarification: The design of this study, data analysis, extraction of key conclusions, and interpretation of results were all independently completed by our research team, without the use of any AI tools to generate text content directly. In order to improve the accuracy of language expression, we only use Grammarly for language refinement in the final revision stage, which includes correcting grammar errors, optimizing sentence structure, and standardizing terminology. Grammarly is a widely accepted tool for grammar and language enhancement and does not generate content. All refinements were based on the original text provided by the authors, ensuring that the core academic content remained unaffected.

To further demonstrate the originality of our manuscript, we can provide relevant materials from the research process, including the original code used for data analysis and multiple draft revisions of the manuscript. These materials clearly illustrate the development and creation process of the paper.

Partial original code for data analysis:

Model 1

help(package="mediation")

library(mediation)

b <- lm(gdm~alt1, data=zong)

c <- lm(lga~alt1+gdm, data=zong)

contcont <- mediate(b, c, sims=50, treat="alt1", mediator="gdm")

summary(contcont)

plot(contcont)

Model 2

library(mediation)

b <- lm(gdm~alt1+age+bmi3+weightgain+gwod, data=zong)

c <- lm(lga~alt1+gdm+age+bmi3+weightgain+gwod, data=zong)

contcont <- mediate(b, c, sims=50, treat="alt1", mediator="gdm")

summary(contcont)

plot(contcont)

Model 3

library(mediation)

b <- lm(gdm~alt1+age+bmi3+weightgain+gwod+g1+mc+pih+qiangdm, data=zong)

c <- lm(lga~alt1+gdm+age+bmi3+weightgain+gwod+g1+mc+pih+qiangdm, data=zong)

contcont <- mediate(b, c, sims=50, treat="alt1", mediator="gdm")

summary(contcont)

plot(contcont)

Multiple version revision records of paper writing:

The first draft of the manuscript was written in Chinese, starting on September 12, 2024, and completed on September 17, 2024. The second draft, also in Chinese, was started on September 19, 2024, with the addition of stratified analysis in the results section and completed on September 22, 2024. The third draft was written in English, translating the previous version, starting on September 22, 2024, and completed on September 23, 2024.

Once again, I sincerely appreciate the reviewer’s valuable feedback. If there are still any doubts regarding certain parts of the manuscript, I am more than happy to provide additional details or explanations to clear up any misunderstandings.

**Comment 2:**

The method section is incompletely written. The inclusion and exclusion criteria are not mentioned.

**Response:**

Thank you for pointing this out. We have now explicitly stated the inclusion and exclusion criteria in the methods section.

Inclusion Criteria: single live birth patients; patients who underwent liver function tests in early pregnancy (11-13 weeks); patients who underwent a 75g oral glucose tolerance test (OGTT) between 24-28 weeks of pregnancy.

Exclusion criteria: patients with unknown gestational age or birth weight; patients with multiple pregnancies; patients with viral hepatitis.

**Comment 3:**

In the results section, Table 1, two very similar numbers in maternal age and age at delivery have significant value. Please check the data and if possible, request the authors to send the data sheet.

**Response:**

Thank you for your careful review and valuable feedback. We have rechecked the data for maternal age and age at delivery in Table 1.

In our study, maternal age and delivery gestational week were analyzed as continuous variables. After performing normality tests, we found that these two variables were not normally distributed, so we used the median and interquartile range for description and conducted a Mann-Whitney U test for group comparisons.Therefore, the similar numbers in Table 1 between the two groups represent the median (interquartile range). However, due to the large sample size and the different distributions of these two variables between the groups, a significant statistical difference was observed between the groups.

To ensure accuracy, we have thoroughly reviewed our dataset and confirmed that the values are correct. Additionally, we have referenced relevant literature in the manuscript to provide further clarification. If necessary, we are happy to provide additional details on the data processing steps. Please let us know if further clarification is needed.

Reference:

- Hart A. Mann-Whitney test is not just a test of medians: differences in spread can be important. BMJ-BRIT MED J. [Comparative Study; Journal Article]. 2001 2001-8-18;323(7309):391-3. doi: 10.1136/bmj.323.7309.391

**Comment 4:**

In conclusion part how the authors conclude “that reducing ALT levels in early pregnancy can help decrease the incidence of GDM as well as the likelihood of delivering LGA infants. “Based on the method and results this conclusion may not be appropriate.

**Response:**

Thank you for your insightful comment regarding the conclusion. We agree that the direct conclusion that "reducing ALT levels in early pregnancy can help decrease the incidence of GDM as well as the likelihood of delivering LGA infants" may not be fully supported by our current data. While our results suggest a potential association between elevated ALT levels in early pregnancy and an increased risk of GDM and LGA infants, we acknowledge that causality cannot be firmly established due to the study's observational nature. Therefore, we will revise the conclusion to more accurately reflect the study design and findings, and provide a more cautious interpretation of the results. We will revise the manuscript accordingly to clarify this point.
